# No unexpected CRISPR-Cas9 off-target activity revealed by trio sequencing of gene-edited mice

**DOI:** 10.1371/journal.pgen.1007503

**Published:** 2018-07-09

**Authors:** Vivek Iyer, Katharina Boroviak, Mark Thomas, Brendan Doe, Laura Riva, Edward Ryder, David J. Adams

**Affiliations:** Wellcome Sanger Institute, Wellcome Genome Campus, Hinxton, Cambridge, United Kingdom; University of California Berkeley, UNITED STATES

## Abstract

CRISPR-Cas9 technologies have transformed genome-editing of experimental organisms and have immense therapeutic potential. Despite significant advances in our understanding of the CRISPR-Cas9 system, concerns remain over the potential for off-target effects. Recent studies have addressed these concerns using whole-genome sequencing (WGS) of gene-edited embryos or animals to search for *de novo* mutations (DNMs), which may represent candidate changes introduced by poor editing fidelity. Critically, these studies used strain-matched, but not pedigree-matched controls and thus were unable to reliably distinguish generational or colony-related differences from true DNMs. Here we used a trio design and whole genome sequenced 8 parents and 19 embryos, where 10 of the embryos were mutagenised with well-characterised gRNAs targeting the coat colour Tyrosinase (*Tyr*) locus. Detailed analyses of these whole genome data allowed us to conclude that if CRISPR mutagenesis were causing SNV or indel off-target mutations in treated embryos, then the number of these mutations is not statistically distinguishable from the background rate of DNMs occurring due to other processes.

## Author summary

The ability to precisely modify the genome has immense therapeutic potential and also represents a powerful tool to understand gene function. One of the key gene editing tools is CRISPR (**C**lustered **R**egularly **I**nterspaced **S**hort **P**alindromic **R**epeats). Although CRISPR can be used to generate a range of genomic alterations or to correct disease causing mutations, concerns over unwanted alterations introduced by poor editing fidelity, so-called “off-targets”, have tempered the use of this technology. Here we use whole genome sequencing to show that off-target mutations are rare and not distinguishable in frequency when compared to naturally occurring mutations.

## Introduction

CRISPR-Cas9 technologies have transformed genome-editing of experimental organisms and have immense therapeutic potential. Despite significant advances in our understanding of the CRISPR-Cas9 system, concerns remain over the potential for off-target effects. Recent studies have addressed these concerns using whole-genome sequencing (WGS) of gene-edited embryos or animals to search for *de novo* mutations (DNMs), which may represent candidate changes introduced by poor editing fidelity[1–3]. Critically, these other studies used strain-matched, but not pedigree-matched controls (i.e. the parents of sequenced offspring were themselves not sequenced) and thus were unable to reliably distinguish generational or colony-related differences from true DNMs. Here we assessed the impact of colony variation on the accuracy of detection of off-target CRISPR editing events.

## Results and discussion

**Fig 1:**
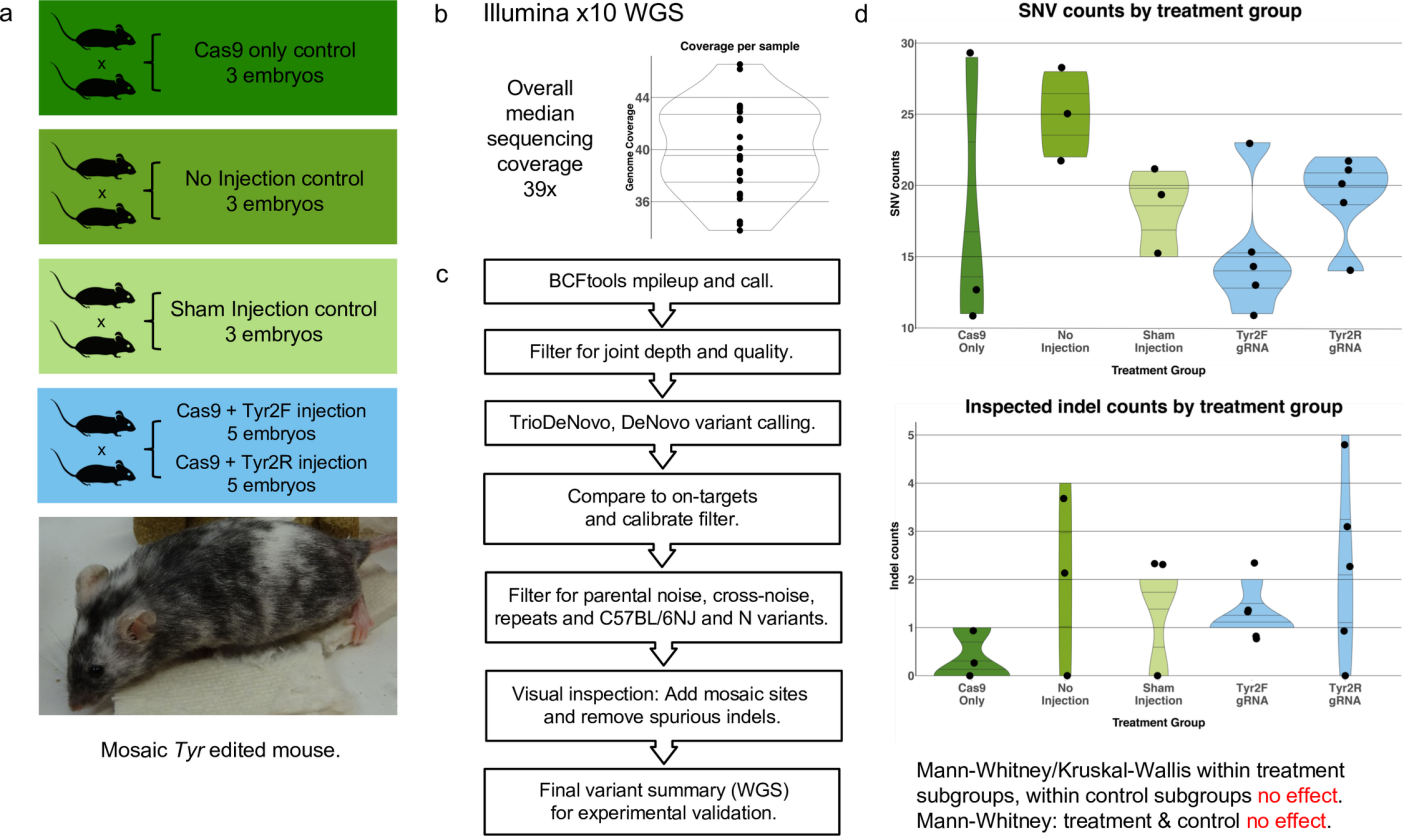
Analysis of CRISPR off-targets by whole genome sequencing. (a) Experimental design: Four sets of C57BL/6N parents gave rise to 9 control embryos (3 “no injection”, 3 “sham injection” with water only and 3 “Cas9 only”), and 10 treated embryos (5 were injected with Cas9 and Tyr2F gRNA and 5 were injected with Cas9 and Tyr2R gRNA). (b) Whole genome sequencing: All 27 mice / embryos were subjected to whole genome sequencing with median depth 39.5x and an average of 3.4% of bases with read depth less than 11x. (c) Variant calling and filtering: starting from the joint variant call (*bcftools mpileup + bcftools call*), a sequence of filter steps were performed to detect *de novo* mutations and remove likely false positives arising from low-level parental mosaicism and alignment errors at repeat regions. Parental-noise: alternate-allele reads present in either parent. Cross-noise: alternate reads from all other (non-parental) samples. (d) Filtered SNV and Indel counts are not significantly different within control groups, within treatment groups, or between control and treatment groups.

In this study, we used a trio design and whole genome sequenced 8 parents and 19 embryos, where 10 of the embryos were mutagenised with well-characterised gRNAs targeting the Tyrosinase (*Tyr*) locus (**Fig 1A**). *Tyr* is responsible for black coat colour and eye pigmentation in C57BL/6 mice[4], so its disruption should not be detrimental to embryonic development. We chose two gRNAs targeting exon 2 of *Tyr*, Tyr2F and Tyr2R, with 395 and 1,502 total predicted off-targets respectively (Methods). The CRISPR-treated group was split to include five embryos treated with Tyr2F and five embryos treated with Tyr2R, while three untreated embryos from each of three control groups (“Cas9 only”, “No injection” and “Sham injection”) were also collected. Microinjections were performed into the cytoplasm of 1-cell zygotes[5], which were then briefly cultured to assess viability and then transferred into 0.5 day post coital (d.p.c) pseudopregnant females. Embryos in the “Sham injection” group were microinjected with water only, and the “Cas9 only” embryos were microinjected with Cas9 protein solution only. All embryos were harvested at 12.5 d.p.c (S1 Table) and genomic DNA from both parents and embryos extracted. Sequencing was performed on the Illumina X10 WGS platform yielding a median sequencing depth of 39.5x per genome (**Fig 1B**, S2 Table). In parallel, targeted Illumina MiSeq sequencing to a mean depth of 10,800 reads of the *Tyr* target site was performed to comprehensively profile mosaicism, with these data analyzed using the CRISPResso software[6] (S3 Table). MiSeq analysis revealed a targeting efficiency of 88% for Tyr2F and 91% for Tyr2R and mosaicism, with a median of two variants per embryo (S3 Table). Importantly, to ensure our experiment was representative of the many thousands of CRISPR experiments performed worldwide, including those of the International Mouse Phenotyping Consortium, we compared these data to MiSeq data from 324 mice mutagenized using the Tyr2R gRNA, revealing good concordance of targeting efficiency and mosaicism (S3 Table).

We next performed variant calling on the WGS data using *bcftools mpileup* and *bcftools call*[7], configured to be sensitive to low allele-fraction indels (insertions/deletions)(**Fig 1C**). We started with a median of 324,561 variants per sample (S4 Table). Candidate DNMs (those not inherited from either parent) in the embryos were called using the TrioDeNovo software[8], which resulted in a median of 7,460 unfiltered DNMs per embryo.

We first looked for the presence of these unfiltered DNMs within 10bp of any potential CRISPR off-target site for Tyr2F or Tyr2R, as determined by the Cas-OFFinder software[9] with up to 3 mismatches with a 1 nucleotide DNA/RNA bulge or up to 4 mismatches without a DNA/RNA bulge. Importantly, we found no such coincident sites in any embryos from the CRISPR-untreated group and only on-target variants in the CRISPR-treated group (S5 Table), suggesting that if there are recurrent CRISPR-induced off-target alterations they are exceedingly rare. When we extended the number of mismatches up to 7 nucleotides (without a DNA/RNA bulge), we did find between 4 and 14 coincident sites per animal, but there was no significant difference in the number of such sites between CRISPR-treated and untreated groups (S5 Table, Methods). In fact, given the total fraction of bases at each possible off-target site and the number of candidate DNMs, we expected to find approximately 32 intersections between our candidate DNMs and the expected off-target positions based on chance alone (Methods). This is higher than the observed intersection counts, but is of the same order of magnitude.

We then filtered the original calls to ensure adequate depth (10x) and a variant quality of 10, in all samples, resulting in a median of 225,671 variants per sample and a median of 6,852 TrioDeNovo called DNMs per embryo (489 SNVs / 6,450 indels) (S4 Table). We next applied a validated filtration strategy to refine our candidate DNM calls[10], removing false positives arising from mosaic alleles in either parent, as well as those in proximity to repeats. Alignments for all SNVs and indel variants were then inspected visually for the presence of mosaic alleles (i.e. a second alternative allele at the same locus). In the same way, all indels were visually inspected to remove further false positives. This resulted in a median of 19 SNVs and 1 indel per embryo (Table 1), which is broadly consistent with prior work that aimed to classify and estimate the *de novo* mutation rates in mice[10]. All variants were validated using targeted MiSeq sequencing to a depth of at least 10,000 reads per locus (S8 Table), yielding an 87% validation rate for SNVs and a 73% validation rate for indels. This is lower than comparable rates from the Mouse Genomes Project[11], but this is expected, given the lower variant allele fraction of our variants. These rates are broadly comparable to validation rates for somatic variants called from cancer genomes.

**Table 1:** Initial and filtered *de novo* variant counts from whole genome sequencing. Summary table of initial variant counts, *de novo* variant counts, and filtered SNVs and Indels. Treatment Groups: Cas9 only (without gRNA), no injection (uninjected embryos), sham injection (water only), Tyr2R treated (Tyr2R gRNA + Cas9), Tyr2F treated (Tyr2F gRNA + Cas9). Variants passing basic depth / quality filters: *bcftools* joint call variant count per animal passing joint-depth and genotype quality filters (see Methods). Candidate *de novo* mutations: all candidates produced by TrioDeNovo caller. Final SNVs / Final Indels: all SNVs/Indels remaining after filtering for false positives arising from low-level mosaicism, known C57BL/6NJ & C57BL/6N variants, proximity to UCSC repeat regions and further visual inspection.

Mouse Sample	Treatment Group	Relationship	Variants passing Basic depth / Quality Filters	Candidate *de novo* Mutations	Final SNVs	Final Indels
MD5617a	-	parent	225,408	-	-	-
MD5618a	-	parent	229,105	-	-	-
MD5630a	cas9 only	embryo	226,692	7,106	13	0
MD5631a	cas9 only	embryo	216,975	6,533	11	0
MD5632a	cas9 only	embryo	217,421	6,626	29	1
MD5619a	-	parent	226,236	-	-	-
MD5620a	-	parent	230,156	-	-	-
MD5624a	no injection	embryo	228,022	7,377	25	4
MD5625a	no injection	embryo	219,537	6,599	22	2
MD5626a	no injection	embryo	217,827	6,510	28	0
MD5616a	-	parent	224,201	-	-	-
MD5623a	-	parent	221,461	-	-	-
MD5627a	sham injection	embryo	226,709	7,732	15	2
MD5628a	sham injection	embryo	225,793	7,693	21	2
MD5629a	sham injection	embryo	224,692	7,646	19	0
MD5621a	-	parent	228,855	-	-	-
MD5622a	-	parent	228,994	-	-	-
MD5633a	Tyr2R treated	embryo	228,026	7,083	19	0
MD5634a	Tyr2R treated	embryo	225,671	6,837	14	3
MD5635a	Tyr2R treated	embryo	231,888	7,308	22	5
MD5636a	Tyr2R treated	embryo	226,002	6,985	21	2
MD5637a	Tyr2R treated	embryo	221,699	6,618	20	1
MD5638a	Tyr2F treated	embryo	222,030	6,852	15	1
MD5639a	Tyr2F treated	embryo	219,136	6,440	13	2
MD5640a	Tyr2F treated	embryo	220,946	6,750	11	1
MD5641a	Tyr2F treated	embryo	222,174	6,803	14	1
MD5642a	Tyr2F treated	embryo	226,899	7,000	23	1
**MEDIAN**			**225,671**	**6,852**	**19**	**1**

A comparison of the expected variants at the *Tyr* locus detected by WGS and targeted MiSeq sequencing (S6 Table) shows that of the 20 indels detected by the MiSeq pipeline, 18 were also detected by the WGS pipeline, with the missing indels having low allele frequencies (7% and 7.5%, as defined by MiSeq sequencing). We are therefore confident that our WGS pipeline will detect genome-wide off-target damage with a range of allele fractions and mosaicism similar to on-target variants. Our median on-target variant allele fraction is 0.28 (S6 Table, column I), corresponding to a heterozygous mutation occurring at the two-cell embryo stage. Given our median depth (39.5x) and our minimum required *de novo* allele frequency (10%, Methods), our expected power to detect a DNM occurring in the single-cell or two-cell stage of the zygote is predicted to be at least 99.5%.

Using the final counts of filtered SNVs and indels for each embryo (**Table 1**), we conducted a Kruskal-Wallis Rank test, detecting no significant difference in DNM counts between the “no injection”, “sham” and “cas9 only” untreated embryo groups (p = 0.30 and p = 0.37 for SNVs and indels, respectively). Similarly, a Wilcoxon Rank Sum test failed to detect significantly different SNV- or indel- DNM counts between the “Tyr2F” and “Tyr2R” CRISPR-treated groups (p = 0.25 and p = 0.43 for SNVs and indels, respectively). Based on these analyses (**Fig 1D**), we combined variant calls from embryos in the two CRISPR-treated groups and in the same way combined data from the three untreated groups. Notably, using these data a Wilcoxon Rank Sum test failed to detect a significant difference in SNV or indel counts between the CRISPR-treated and untreated groups; p = 0.30 and p = 0.45, respectively (S7 Table).

We also measured the impact of using unrelated parents on the false-positive DNM rate by deliberately choosing the parents of the Cas9-only embryos when analyzing all embryos in the study; the male parent (CBLT8902) was greater than five generations removed from all other male parents (S1 Fig). Performing a comparable subset of filtrations and comparing variant counts by sample to the correctly analysed embryos at the same filtration point showed a median increase of 66 false variants per embryo (S1 Fig, S9 Table), highlighting the importance of using trios of mice when studying potential off-target rates.

Finally, to investigate the robustness of this result, we reanalyzed all of our genome sequencing data using a combination of two somatic variant callers (searching for SNVs with CaVEMan[12] and indels with cgpPindel[13]). These callers were used to search for SNVs and indels present in the treated and untreated embryos that were not present in the parents. The caller output was subject to an analogous filtering pipeline (Methods). Although not designed to search for DNMs, somatic variant callers can be more sensitive to low variant allele fraction mutations. Indeed, we detected more filtered mutations with this approach than with TrioDeNovo (S10 Table, S11 Table). However, identical statistical tests on the final filtered counts of SNVs and indels (Methods, S12 Table) again failed to detect any significant differences between gRNA-treated and untreated groups.

We conclude that if CRISPR mutagenesis performed under the conditions we have described were causing SNV or indel off-target mutations in treated embryos, then the number of these mutations is not statistically distinguishable from the background rate of DNMs occurring due to other processes. This work should support further efforts to develop CRISPR-Cas9 as a therapeutic tool.

## Materials and methods

### Ethics statement

Following approval by the Sanger Institute Animal Welfare and Ethical Review Body (AWERB) all procedures were performed at Wellcome Trust Sanger Institute Research Support Facility under Home Office licensed authority, Establishment Licence Number X3A0ED725, Project licence number P96810DE8. The use of animals in this study has been carried out in accordance with the UK Home Office regulations under the Animals (Scientific Procedures) Act 1986.

### 1. gRNA choices and characterisation of efficiency and mosaicism

We chose two gRNAs (Tyr2F and Tyr2R) within exon 2 of the Tyrosinase (*Tyr*) gene, as mutations at this locus do not cause lethality or influence embryogenesis. *Tyr* is responsible for black coat colour and eye pigmentation in wild type (WT) C57BL/6 (B6) mice and bi-allelic mutation of this gene results in complete loss of pigmentation (albinism)[4]. Therefore, detection of biallelic mutations caused by CRISPR/Cas9 activity is easily visible as a change in coat colour.

The Tyr2R gRNA was selected as previously described[14] and together with the Tyr2F gRNA represent a typical range of off-target scores. Off-target scores for the Tyr2F and Tyr2R gRNAs were determined for the default (NGG) PAM only, using the reference mouse genome (mm10) in the Cas-OFFinder tool[9] (Table 2). Off-target scores were also determined for the Schaefer sgRNA#4[15] using the FVB/NJ mouse genome, to ensure that off-target scores were similar. Whilst we are not trying to directly compare our results to the Schaeffer paper [3], we feel that it is important to demonstrate that the off-target scores for our gRNAs are not significantly different to their study. The sequences for the gRNAs used in this experiment are shown in Table 3.

**Table 2:** Summary of off-target scores for selected gRNAs. Off-targets numbers as determined by Cas-OFFinder [9] for Tyr2F, Tyr2R used in our experiment and Schaeffer (15).

CRISPR	Sequence	Number of nucleotide mismatches					Off-target totals
		0	1	2	3	4	
Tyr2F	Target only	1*	0	0	4	51	55
	Target +1 (DNA bulge)	0	3	5	75	-	83
	Target -1 (RNA bulge)	2	2	8	245	-	257
							**395**
Tyr2R	Target only	1*	0	2	15	161	178
	Target +1 (DNA bulge)	1	1	12	324	-	338
	Target -1 (RNA bulge)	0	4	71	911	-	986
							**1,502**
Schaefer sgRNA#4	Target only	1*	0	0	10	129	139
	Target +1 (DNA bulge)	0	2	8	226	-	236
	Target -1 (RNA bulge)	1	3	55	923		982
							**1,357**

* This indicates the *on-target* gRNA

**Table 3:** Sequences for Tyr2R and Tyr2F gRNAs.

gRNA label	Sequence (PAM in bold)
Tyr2R	GCTCCCATCTTCAGCAGATG**TGG**
Tyr2F	TTTCCAGGATTACGTAATAG**TGG**

To determine the expected cutting efficiency and mosaicism from a large number of experiments we chose Tyr2R, comparing different Cas9 sources (mRNA or protein) as well as gRNA sources (*in vitro* transcribed or synthetic). The gRNA was mixed in RNase free water (Ambion) at a concentration of 25ng/ul together with either Cas9 mRNA or protein at 50ng/ul. The CRISPR reagents were injected into the cytoplasm of zygotes and F0 pups were scored for black, mosaic and albino coat colour. In addition, genomic DNA from earclips was extracted from F0 mice as described in section 3, and MISEQ sequencing analysis performed as described in section 4. This enabled us to determine the targeting efficiency and how many different alleles were present within each founder animal, therefore making it possible to score mosaicism (more than one mutated allele detected in the animal) for each condition. The synthetic gRNA was the most efficient, with 70% of pups showing a mutant genotype. Synthetic gRNAs were therefore used for the trio experiment (S3 Table).

For the trio experiments, synthetic gRNA consisting of crRNA and tracrRNA (Sigma) were diluted and mixed in RNase free water at equimolar ratios of 0.7pmol/ul each. Cas9 protein (obtained from Marko Hyvonen, Department of Biochemistry, University of Cambridge) was added to a working concentration of 50ng/ul and the mixture was incubated at 25°C for 10 minutes before zygote injection. The concentrations of reagents injected for each of the experimental groups are shown in Table 4.

**Table 4:** Concentration of reagent injected for each experimental group.

Group label	Concentration of reagents injected
‘No injection’ group	No injection
‘Sham injection’ group	RNase free water
‘Cas9 only’ group	50ng/ul Cas9 protein
‘Tyr2R’ and ‘Tyr2F’ group	0.7pmol/ul of crRNA and tracrRNA, 50ng/ul Cas9 protein

DNA was extracted as described in section 3 and MISEQ sequencing analysis performed as described in section 4. The results are presented in S3 Table labelled with the treatment condition (e.g. ‘No injection’, etc), and are used to check that the trio experiment is comparable to historical data at the target locus.

### 2. Zygote injection

4 x 4-week old C57BL/6NTac females were super-ovulated by intraperitoneal (IP) injection of 5 IU of pregnant mare’s serum (PMSG) at 11.00hrs (12hr light/dark cycle, on at 07:30/off at 19:30) followed 48hrs later by an IP injection of 5 IU human chorionic gonadotrophin (hCG) and mated overnight with C57BL/6NTac stud males. The next morning females were checked for the presence of a vaginal copulation plug as evidence of successful mating and females housed separately, noting which males were used to plug each of the females. Oviducts were dissected one female at a time and harvests of cumulus masses kept separately in 4 different groups. Cumulus masses were released and treated with hyaluronidase as previously described[16]. Fertilized 1-cell zygotes, confirmed by the presence of 2 pronuclei, were selected and maintained in KSOM media prior to cytoplasmic injection at 37°C in 4 separate dishes. Microinjections were carried out between 23–25hrs post hCG. Although the exact cell cycle stage varied from zygote to zygote, microinjections were done prior to coalesence of the pronuclei, and therefore the completion of S-phase. Cytoplasmic Injections were carried out as in S1 Table. The tyrosinase Tyr2F and Tyr2R microinjected embryo groups both came from the same zygote pool and hence had the same parentage. All other microinjection groups had a unique set of parents.

The Cas9 ribonucleoproteins (RNP) were backfilled into a microinjection needle. Microinjections were carried out using positive balancing pressure, microinjecting into the cytoplasm of fertilized 1-cell zygotes held in FHM medium. A successful injection was indicated by visible movement in the cytoplasm after breaking the Oolemma. Microinjected 1 cell embryos were briefly cultured and viable zygotes were transferred the same day by oviducal embryo transfer into a 0.5 days post coital (d.p.c.) pseudo-pregnant female F1 (CBA/C57BL/6J) recipients[16]. After 12.5 d.p.c. recipient mice were humanely culled and embryos dissected and snap frozen. Previously, the parents from each group were humanely culled, tissue taken and labelled according to which microinjection group they contributed to (S1 Table).

All procedures performed in studies involving animals were in accordance with the ethical standards of the institution or practice at which the studies were conducted and performed with approval of the UK Home Office.

### 3. DNA extraction from mice and embryos

For the historical data, genomic DNA was extracted from earclips of F0 mice using the Sample-to-SNP kit lysis buffer (Life Technologies).

For the trio experiments, genomic DNA was extracted from the kidney of parent animals or from macerated whole 12.5 d.p.c. embryos using the DNeasy Blood & Tissue Kit (Qiagen) according to manufacturer's instructions. DNA was quantified using a NanoDrop spectrophotometer.

Note that for the historical data, extracting DNA from ear-clips could bias the estimate of on-target mosaicism, depending on the distribution of mutant cells. The mosaicism rate from historical data is, however, approximately the same as the current mosaicism rate, with approximately 2 on-target variants per embryo (S3 Table). Similarly, the choice to extract DNA from parental kidneys only could bias the *de novo* variant count for particular pedigrees. However, any bias in a single pedigree should be mitigated by the use of three control groups from different pedigrees. Additionally, the high sequencing depth used when validating all candidate *de novo* variants would minimise possible bias resulting from subclonal mutations in the parental kidneys.

### 4. Amplicon sequencing and analysis at the *Tyr* locus of mice and embryos

This analysis was performed on 324 historical mice, and on the parents and embryos in the trio experiment. 1μl of genomic DNA was used for amplicon specific PCR using genome specific primers (PE_tyrex2N_F1 and PE_tyrex2N_R1, Table 5), which flanked the expected on-target sites for Tyr2R and Tyr2F. The indexed libraries were sequenced using standard protocols and Illumina MiSeq technologies (Paired End 250bp runs).

**Table 5:** Sequences of primers flanking expected on-target sites for Tyr2R and Tyr2F.

**PE_tyrex2N_F1**	**ACACTCTTTCCCTACACGACGCTCTTCCGATCTACCTTCCAGTGTGTTTCTAAAGC**
**PE_tyrex2N_R1**	**CGGTCTCGGCATTCCTGCTGAACCGCTCTTCCGATCTAACTGCCAGAAAGCTGAATGA**

The paired-end fastq files generated were analysed using the CRISPResso sequence analysis package[6]. A typical command line used to run CRISPResso specified both gRNA sequences concurrently and allowed a window of 30bp around the gRNA sites to detect possible mutations: *CRISPResso -r1 sample_read1*.*fastq*.*fastq -r2 sample_read2*.*fastq -a <unedited_amplicon_sequence> -w 30 -g <Tyr2R_sequence>*,*<Tyr2F_sequence>*. The resulting sequence variants were visually inspected for indels with an allele fraction of at least 5%.

MiSeq analysis performed on the target region in these samples using CRISPResso showed that the parents, no injection, sham and Cas9 only groups showed no on-target activity, whereas the two gRNAs showed efficient cutting at their target site (S3 Table). Of the 10 mice showing targeting for the Tyr2R gRNA, 3 had one allele (30%), 5 had two alleles (50%) and 2 had three alleles (20%). Of the 7 mice showing targeting for the Tyr2F gRNA, 4 had two alleles (57%), with the remaining three mice containing either one, three or four alleles. This is in broad concordance with the historical data (S3 Table).

In order to cover a representative range of alleles at the on-target site, we selected the following mice for WGS analysis: for the Tyr2R sample 1 mouse with one allele, 3 mice with two alleles and 1 mouse with three alleles; for the Tyr2F sample 1 mouse with one allele, 3 mice with two alleles and 1 mouse with three alleles (in total five embryos Tyr2F and five embyros for Tyr2R).

### 5. Whole genome sequencing

Whole genome sequencing libraries were prepared using standard protocols for the Illumina X10 platform (ENA Accession ERP024425). The resulting sequence was aligned using *bwa mem* to the reference mouse GRCm38 assembly. The total mapped coverage varied from 34x to 47x, with a median of 39.5x (Fig 1B). The median fraction of bases with a coverage of greater than 50x was 39% and the median fraction of bases with a coverage of less than 11x was 3.4% (S2 Table).

### 6. Probability of detecting de novo mutations at low allele fractions (power)

Our median observed on-target variant allele fraction was 0.28 (median of column I, S6 Table). This allele fraction is represented by 11 variant reads out of 39.5 reads, and most likely arises from a heterozygous mutation in the two-cell embryo. We take this frequency as a representative effect size. To find our power to resolve mosaic DNMs from our WGS data with (median) 39.5x read coverage for this effect size, we modelled the actual number of mutant reads observed with a Poisson distribution with parameter *lambda* = 11. Since our minimum required variant allele frequency is 10% (Methods Section 7), we must observe at least 4 mutant allele reads out of 39 to call a DNM. The probability of *not* calling a mutation—seeing 3 or fewer reads—in this case is given by the R function *ppois(3*,*11*,*lower*.*tail = TRUE)* = 0.005. This suggests that when we sequence our 12.5dpc embryos, we should detect DNMs, which occur in a two-cell embryo (or earlier) at least 99.5% of the time.

### 7. Variant calling, trio analysis pipeline and filtering of candidate *de novo* mutations

Aligned sequence was jointly variant called for all parents and offspring using *bcftools mpileup*, *bcftools call*, *bcftools norm and bcftools filter*[7]. The *bcftools* version and command options used are as follows: *bcftools-1*.*6 mpileup -a AD -C50 -pm2 -F0*.*05 -d10000*, *bcftools-1*.*6 call -vm*, *bcftools-1*.*6 norm -m -any*, *and bcftools-1*.*6 filter -m+ -sLowQual -e"%QUAL< = 10" -g3 -G10*.

Note: a joint (multiallelic) variant call was performed on all parents and offspring at the same time, using *bcftools mpileup* configured for sensitivity (requiring only 2 or more indel reads and a minimum indel allelic fraction of 0.05). *bcftools* called between 317,230 and 332,048 variants per sample with a genotyping quality > = 10, with a median of 324,561 variants (S4 Table). All variant loci were required to have a total depth of at least 10 reads for further analysis.

*De novo* mutation calling on all variants was performed by running TrioDeNovo software[8], using default settings, independently on each parent/offspring trio. After filtering for depth and quality as above, TrioDeNovo produced between 6,437 and 7,732 candidate mutations per offspring; with a median of 6,852 mutations (S4 Table).

Based on the read depths and variant allele fractions seen in the expected on-target mutations called by TrioDeNovo, we filtered all candidate *de novo* variants with the following criteria, to remove likely false positives: (1) We required a minimum variant allele fraction of 10% to allow for mosaic alleles. (2) To avoid false positives arising from low-allele-fraction mosaic variants in either parent, we removed variants with *any* alternate-allele reads present in *either* parent (“parental noise”). (3) We removed any variant coincident with an allele reported in the C57BL/6NJ strain of the Mouse Genomes Project[11] or specifically in the C57BL/6N^*Tyr*^ strain[17]. (4) To avoid further false positives arising from low-allele-fraction mosaic variants present in the parents, we allowed a maximum contribution of only 2% alternate reads from all other (non-parental) samples (“cross noise”). (5) As repeat regions can cause mis-alignment of reads resulting in false positive calls, we merged all individual UCSC repeat tracks with the UCSC RepeatMasker track (about 1.2Gb of sequence) and removed any variant inside or 1bp adjacent to any merged repeat region. (6) We removed any *de novo* variant shared by two or more samples, as this would be extremely unlikely, and such mutations are more likely mosaic in the parents. Although it is possible this could remove a preferred off-target mutation, we note this removed only 3 variants. (7) Every variant locus was visually inspected to check whether any position was actually mosaic (i.e. contained two or more alternative variants). These extra alternate variants were not consistently called and had to be manually re-inserted. (8) We found indel variants to be especially susceptible to false positive calls arising from un-annotated microsatellites or repeats, so we visually inspected all indel variants to remove any variant still in or adjacent to microsatellites/homopolymers, which were not annotated by UCSC. These filters resulted in 11 to 29 SNVs per sample (median 19) and 0 to 5 indels per sample (median 1). Every SNV and indel has been listed in S8 Table.

### 8. Intersection of all variants with potential off-target locations for Tyr2F and Tyr2R gRNAs

The CAS-OFFinder tool[9] was used to find all potential off-targets sites based on sequence homology to either the Tyr2F or Tyr2R gRNAs, allowing up to 3 mismatches with 1 bp of inserted or deleted sequence and up to 4 mismatches with no inserted or deleted sequence. This resulted in 395 and 1,502 potential off-target sites being detected for Tyr2F and Tyr2R, respectively (S5 Table; RGEN_Tyr2*_OffTargetSites). These 23bp gRNA positions were intersected (allowing for a window size of 10bp) with *all* candidate DNMs before filtering, using *bedtools-2*.*23 window*. The results are presented in (S5 Table, worksheet “RGEN_DNM_10bp_overlap”).

We investigated the coincidence of DNMs with expected off-target sites containing up to 7 mismatches, including both–AG and–GG PAM sequences. With 276,254 potential off-target sites for the combined Tyr2R and Tyr2F gRNA (Ty2F, 119,754; Tyr2R, 156,500), we expect approximately 32 intersections between these candidate off-target sites and our candidate DNM sites. This is based on an expected 7,460 unfiltered DNMs per animal and a total number of 43 * 276,524 = 6,360,052 bases covered by candidate off-target sites and a mouse genome size of 2.8*10^9 bases: expected intersections = number of attempts * probability of success of intersection = 7,460 * 43 * 276,524 / (2.8*10^9) = 31.6. Using *bedtools-2*.*23 window–w10*, we found between 4 and 14 overlaps per sample (S5 Table, worksheet “RGEN_7mm_overlap_counts”), which is lower, but the same order of magnitude as the estimate. We assessed the null hypothesis that the intersection counts of *Tyr*-treated and *Tyr*-untreated animals were drawn from the same population using a Wilcoxon Rank-Sum test, with the R function *wilcox*.*test*, and found no significant difference in intersection counts between the population of treated animals and the population of untreated animals.

### 9. Comparison of SNVs and indel counts between CRISPR-Cas9 treated and untreated groups of the trio experiment

Due to the small number of samples and the low variant counts in each group, we chose to perform either a Kruskal-Wallis Rank Sum test (in the case of more than two groups) or a Wilcoxon Rank Sum test (for only two groups) to assess the null hypothesis: namely that the counts in each group were drawn from the same population. Four tests were performed using the R function *kruskal*.*test or wilcox*.*test* and a Bonferroni-adjusted critical p-value of 0.0125, comparing within treated or untreated groups for SNVs or indels. These tests were not significant. Finally, a Wilcoxon Rank Sum test compared all untreated to all treated embryos for SNVs or indels. This test was also not significant. The groups compared, the values tested, the tests used and test results are presented in S7 Table.

### 10. Validation of WGS variant calls with PCR and MISEQ sequencing

1μl of genomic DNA was used for amplicon specific PCR using genome specific primers (S8 Table). The indexed libraries were sequenced using standard protocols and Illumina MiSeq technologies (Paired End 250bp runs).

Each candidate position was sequenced to an average depth of at least 10,000 reads in embryos and (pooled) parent samples. The sequences were directly aligned to the GRCm38 assembly using *bwa-mem*, and the resulting alignments directly inspected at each variant location using *samtools-1*.*3*.*1 mpileup -d50000 -Q0 -q0* to check for the presence of the alternate allele in the parents’ sample, and to confirm the alternate allele in the embryo sample. Locations in the pooled parent sample with more than 100 reads showing the alternate allele and greater than 1% alternate allele fraction were classed as not validated, as were locations in the embryo samples showing less than 1% alternate allele fraction. We found that 87% of SNVs and 73% of indels were validated (S8 Table).

### 11. Re-analysis of de novo mutations with genetically distant parents

To estimate the effect of using a distantly related parent on the measured *de novo* mutation rate, we re-analyzed our embryos for DNMs using a genetically distant parent. Specifically, it can be seen from the relationships of matings contributing to our experiment’s parents and embryos (S1 Fig) that the male parent of the “Cas9 only” embryos (mating CBLT8902) was distant by more than five generations from the equivalent male parents of the other treatment groups (matings CBLT8762, CBLT9125, CLBT8712), whereas the female parents for all treatment groups were drawn from mating CBLT9125. We therefore chose to *fix* the parents for the “Cas9 only” group as the parents for *all* treatment groups as input to TrioDeNovo and reran our pipeline, including filtration of variants up to the removal of C57BL/6NJ and C57BL/6N variants as well as repeats (filtration steps 1–3 and step 5, section 7). It was not possible to run further filtration, e.g. removal of cross-animal noise, as the “correct” parents were present in the other animals.

The results show a median of 98.5 DNMs per animal for the treatment groups with distantly related controls (“No Injection”, “Sham injection”, “Tyr2F” and “Tyr2R”), whereas the treatment group with the correct control (“Cas9 Only”) has a median number of 28 DNMs per animal (S1 Fig, S9 Table). The equivalent median of all treatment groups is 32 (S4 Table, column L; ‘not on-target, vaf, parent noise, repeat and BL6NJ/N filtered’). This demonstrates the effect of using unrelated or distantly related parents as controls, when searching for CRISPR off-targets, is an inflation of 66.5 false positive variants and reinforces the need for studies to use trios in these experiments.

### 12. Re-analysis of de novo mutations using somatic variant callers

We investigated the robustness of our results by re-analysing our aligned reads using somatic variant callers developed for the analysis of cancer genomes. We labelled each embryo as a “tumour” and each parent sample as a “normal”. We used CaVEMan[12] to call SNVs and cgpPindel[13] (a modified Pindel version 2.0) to call indels.

CaVEMan called between 1,854 and 3,700 passing variants per sample with a median of 2,845 variants (S10 Table, worksheet “Caveman”). In order to remove false positive variants, we applied the following filters to the CaVEMan calls: (1) ASMD (the median alignment score of reads showing the variant allele) > = 140. (2) Read depth > = 10 for both the embryos and the parents, and alternative read depth > = 4 in the embryos. (3) Variant allele fraction > = 10%. (4) Alternative read depth = 0 in either parent, to avoid false positives arising from low-allele-fraction mosaic variants. (5) Somatic calls had to be present in the comparisons with both parents. (6) Variants could not be shared between two or more samples. (7) Variants could not coincide with an allele reported in any strain of the Mouse Genomes Project[11]. (8) We excluded variants inside or 1bp adjacent to any repeat region (we used UCSC repeat tracks as described above).

Using the default WGS panel of filtering rules [12], CgpPindel produced between 44 and 116 candidate indels per offspring, with a median of 71.5 indels (S10 Table). To this output, we applied the following additional filters. (1) We selected the variants with allele fraction > = 10%. (2) We selected for each embryo only the somatic calls present in the comparisons with both parents. (3) We removed any variant shared by two or more samples. (We note that only one indel is shared between two samples, which are treated with the same gRNA–namely, between MD5639a and MD5642a. However, this shared indel is in a microsatellite region, and it is not predicted to be a CRISPR off-target location). (4) We filtered variants coincident with an allele reported in any strain of the Mouse Genomes Project[11]. (5) We excluded variants inside or 1bp adjacent to any repeat region (we used UCSC repeat tracks as described in the previous section).

After filtering SNVs and indels in this way, we obtained 14 to 36 SNVs per sample (median 21) and 0 to 6 indels per sample (median 2). All 471 SNVs and indels are listed in S11 Table. The somatic calling pipelines identified 80% (313 out of 389) of the variants identified by the *de novo* mutation calling pipeline. The mutations identified by the somatic pipelines, which were not identified by the *de novo* pipeline, had a lower variant allele frequency (median of 12%) compared to those identified by both methods (median of 34%). We also noticed that cgpPindel identified an additional mosaic 337 bp deletion at the Tyr2F on-target location for MD5638a (S6 Table).

As with the previous variant analyses, we performed a Kruskal-Wallis Rank Sum or Wilcoxon Rank Sum tests to assess the null hypothesis that the SNV or indel counts from each of the control groups or treatment groups were drawn from the same population (S12 Table). We performed four such tests and they failed to produce any significant result at the Bonferroni-adjusted critical p-value of 0.0125. Finally, a Wilcoxon Rank Sum test comparing the combined untreated group to the combined treated group, also failed to produce a significant result (S12 Table).

## Supporting information

S1 Fig Analysis of parent effects on *de novo* mutations. (a) Pedigree of heredity between the matings of mice from which mice parents were drawn. All female parents were drawn from mating CBLT9125 (red). Male parents were drawn from matings CBLT8712, CBLT8762 and CBLT8902 (dark blue). CBLT8712, CBLT8762 were greater than five generations distant from mating CBLT8902. (b) Scheme of the filtration pipelines for both the “correct” calling approach and the “incorrect parent” calling approach, generating variant counts that can be compared. (c) Graphs of variants counts from “correct parent” pipeline and “incorrect parent” pipeline showing the effect of choosing distantly related parents on *de novo* variant calls: an average increase of greater than 60 variants.
(TIF)

S1 Table List of zygote injections. Counts of embryos microinjected, transferred and harvested at 12.5 d.p.c by treatment group.
(XLSX)

S2 Table Illumina X10 read coverage. **Worksheet: Total Depth**
Total numbers of bases sequenced by Illumina X10 and average depth by mouse sample.
**Worksheet: Depth by Coverage Bin**
For each sample the columns 1+, 11+ etc show the fraction of all sequenced bases at coverage greater > = 1x, > = 11x, etc.
(XLSX)

S3 Table Summary of mosaicism for determining cutting efficiency. Cutting efficiency and mosaicism obtained from Tyr2R, using different Cas9 sources (mRNA, protein) and different gRNA sources (*in vitro* transcribed or synthetic). Historical cutting efficiency is compared to the current experiment.
**Worksheet: Efficiency and Mosacism**
Shows cutting efficiency and mosaicism rates from both historical mouse data (Rows “Cas9 Protein” and “Tyr2R RNP”) and the present experiment (Rows “parent”, “No Injection”, “Sham injection”, “Cas9 only”, “Tyr2F”, “Tyr2R” describe each treatment condition). Historical data shows broad similarity to current data conditions “Tyr2R” and “Tyr2F” for targeting efficiency (column E) and average variants per embryo (column G).
**Columns:**
Targeting efficiency: the number of animals displaying a mutant genotype with allele frequency > 5%. Percentage is stated of the number of pups analysed.
Number with Mosaic genotype: The number of animals or embryos displaying multiple alleles at the same target site. Percentage is stated of the number of pups analysed.
Average variants per embryo: the average number of distinct alleles per embryo or mouse in each historical or current treatment group.
**Worksheet: TestsOfDifferentConditions**
Historical data. Shows the expected cutting efficiency and mosaicism of Tyr2R under different Cas9 sources and gRNA sources. Synthetic gRNA was most efficient with 57 out of 82 pups born showing indels (70%). On average, 53% of mice showed one allele, 33% two alleles, 12% three alleles, 2% four alleles and 0.5% five alleles. As the synthetic gRNA showed the highest cutting efficiency, we used these for further experiments.
(XLSX)

S4 Table Total variant counts and filtered counts by sample. This is a detailed version of Table 1, with further details on the reduction of variant counts as successive filters are applied. (see Methods). Samples are grouped into parent / offspring groupings.
**Columns:**
Sample: sample label of each parent or embryo
Treatment Group:
Cas9 only—gRNA omitted in injection
No injection—uninjected embryos
Sham injection—injected with water
Tyr2R treated—treated with Tyr2R gRNA + Cas9
Tyr2F treated- treated with Tyr2F gRNA + Cas9
Quality passed: Variants per sample passing basic genotype quality > 10
Depth and quality passed: variant count per animal passing joint-depth (>10) and genotype quality filters
TrioDeNovo, depth, quality: all candidates produced by TrioDeNovo caller passing the previous two thresholds.
TrioDeNovo, depth, quality SNVs / Indels: breakdown of TrioDeNovo column by variant class.
On target: Indel variants produced by TrioDeNovo lying in the expected Tyr mutation regions
Not on-target, vaf and parent noise filtered: all SNVs/Indels *not* in the Tyr locus, remaining after filtering for false positives arising from parental mosaicism and minimum Variant Allele Fraction (> = 0.1)
Not on-target, vaf, parent noise and repeat filtered = Variants passing prior filters which are not in or 1bp adjacent a UCSC repeat region.
Not on-target, vaf, parent noise, repeat and BL6NJ/N filtered = Variants passing prior filters which are not known C57BL/6NJ or C57BL/6N variants.
Not on-target, vaf, parent /cross noise, repeat and BL6NJ/N filtered: Variants passing prior filters which are not present at more than 2% in any other samples.
“… Without shared DNVs”: Variants passing prior filters, which are not shared between any two embryos:
Final SNVs: SNV variants passing all previous filters (and manual re-addition of any mosaic variant at same locus, if it exists).
Filtered Indels: Indel variants passing all previous filters (and manual re-addition of any mosaic variant at same locus, if it exists).
Final indels: indel variants passing all previous filters, visually inspected for correctness.
(XLSX)

S5 Table Off-target locations with adjacent *de novo* mutations. **Worksheets: RGEN_Tyr2F_Offtarget_Sites, RGEN_Tyr2R_Offtarget_Sites: All expected CRISPR off target locations for Tyr2F and Tyr2R.**
Columns state Chromosome, Position and Direction of expected off-target site, as well as the extent of the mismatch (number of basepair mismatch and whether there is a “bulge”—i.e. a 1bp insertion or deletion, and whether the “bulge” is a DNA or RNA bulge).
**Worksheet: RGEN_DNM_10bp_overlap_by_sample**
The intersection (with a 10bp window) of unfiltered *de novo* variant calls with all Tyr2F and Tyr2R gRNA candidate off-target locations. This shows that no untreated animals have any overlap within 10bp of a candidate Tyr2R or Tyr2F off-target site, and that only the on-target indels overlap the expected on-target site in the treated animals. Worksheet: RGEN_7mm_overlap_counts. Counts by sample of the intersection of unfiltered de novo variant calls with candidate Tyr2F/Tyr2R off-target locations (allowing up to 7 mismatches).
(XLSX)

S6 Table WGS and MiSeq on-target alleles for comparison. Shows a comparison of on-target variants revealed by high-depth MiSeq sequencing and CRISPResso analysis (cutoff allele frequency 5%), compared to the variants at the same locations revealed by whole-genome X10 sequencing and *bcftools* analysis. We note that CRISPResso found no on-target variants in the untreated samples (MD5624a-MD5632a), so these samples are not presented. CRISPResso found 20 variants in the *Tyr*-treated variants (MD5633a-MD5642a), with variant allele fractions ranging from 0.1 to 0.33, and with a typical mosaicism of 2 alleles per embryo. This confirms that the *Tyr* gRNAs are active in the treated samples, and that the activity at the on-target locations measured by mosaicism and allele fraction are within expected ranges. Deep-sequencing of the on-target location by MiSeq therefore shows mosaic indels in treated animals, and no mutation in controls. X10 sequencing confirms both location and mosaicism in treated animals, with the exception of three indels, two of which are at low allele fraction (7% and 7.5%), and the third which is a second mosaic allele at *exactly* the same location as a called allele. Our pipeline manually adds in such mosaic alleles.
(XLSX)

S7 Table Results for Kruskal-Wallis and Wilcoxon Rank Sum tests. There are three control groups, containing three samples each: a “no injection” group, a “sham” injection group (injected with water) and a “Cas9-only” group. Due to the small number of samples in each group we elected to perform a Kruskal-Wallis Rank Sum test to detect any differences between these three groups. This test failed to detect any difference in SNV counts between groups–i.e. to reject the null hypothesis (chi-squared = 2.4, df = 2, p-value = 0.3012) and failed to detect any difference in indel counts between groups (chi-squared = 1.9874, df = 2, p-value = 0.3702). The Tyrosinase-treated groups were either Tyr2R-treated (5 samples) or Tyr2F-treated (5 samples). We performed a Wilcoxon Rank Sum test and again failed to detect any difference between the groups for SNV counts (W = 6.5, p-value = 0.2492) or indel counts (W = 8.5, p-value = 0.4338). Finally, based on these results, we merged all three untreated groups together and both *Tyr*-treated groups together. Using the Wilcoxon Rank Sum test, we were unable to detect any difference in SNV counts between the Tyr-untreated and Tyr-treated groups (W = 58, p-value = 0.3059) or indel counts (W = 35.5, p-value = 0.4471).
(XLSX)

S8 Table Filtered variant positions for experimental validation. This lists the genomic location of all final DNM variants (SNVs and Indels) passing all filters and subsequently sent for validation, as well as PCR primer pairs used for validation, and the validation test results.
(XLSX)

S9 Table Filtered variant counts for alternative *de novo* variant analysis. Lists filtered variant counts for an alternative *de novo* variant analysis in which the parents of the Cas9-only trio were deliberately set to be the parents for *all* embryos. *De novo* variant calling and filtration was carried out as before, with filtration stage stopped after the removal of parental mosaicism, proximity to repeats and the overlap with known C57BL/6NJ or C57BL/6N variants. (This is the last sensible filter stage suitable for a comparison of this approach with the original data.) Column G lists the combined SNV and indel counts at this filtration point. As expected, the “Cas9 only” group—which was analysed using the correct parents—has significantly lower values of variants than the other groups. Column G is directly comparable with S4 Table Column L (“not on-target, vaf, parent noise, repeat and BL6NJ/N filtered”).
(XLSX)

S10 Table Total somatic caller variant counts and filtered counts by sample. **Worksheet: CaVEMan:**
This shows the counts of variants arising from somatic callers as successive filters are applied. (see Methods). Samples are grouped into parent / offspring groupings.
**Columns:**
Sample: sample label of each parent or embryo
Treatment Group:
Cas9 only—gRNA omitted in injection
No injection—uninjected embryos
Sham injection—injected with water
Tyr2R treated—treated with Tyr2R gRNA + Cas9
Tyr2F treated- treated with Tyr2F gRNA + Cas9
CaVEMan calls vs P1, CaVEMan calls vs P2: passing variants from the cgpCaVEMan pipeline, from setting the “tumour” sample to be the embryo and the “normal” to be the first and second parent, respectively.
ASMD > = 140 P1, ASMD > = 140 P2: all candidates from the previous step, filtered for the ASMD field > = 140. ASMD is defined as the median alignement score of reads showing the variant allele
CaVEMan filtered calls coverage and alt read vs P1, CaVEMan filtered calls coverage and alt read vs P2: previous calls filtered for total read depth > 10 and alternative read depth > 4.
CaVEMan filtered calls vaf> = 0.1 P1, CaVEMan filtered calls vaf> = 0.1 P2: previous calls filtered to require Variant Allele Fraction (VAF) > 0.1
CaVEMan filtered calls alt in P1 = 0, CaVEMan filtered calls alt in P2 = 0: previous calls filtered to remove any variants showing an alternate-allele read in either parent.
Common calls: previous calls, only keeping those variants called as “somatic” in the embryo for both P1 and P2.
… without shared snvs: previous calls, with any SNVs removed if they were shared between two animals.
Known SNPs filtered: previous calls removing any allele which coincides with any reported allele in the Mouse Genomes Project[11].
SNVs in repeats filtered, final SNVS: previous calls removing any SNV in or 1bp adjacent) a UCSC repeat.
**Worksheet: Pindel**
Sample, Treatment and relationship columns as for previous worksheet.
Pindel calls vs P1, Pindel calls vs P2: passing output from cgpPindel[13].
Pindel filtered calls vaf> = 0.1 P1, Pindel filtered calls vaf> = 0.1 P2, Common calls, … without shared indels, known indels filtered, indels in repeats filtered, final indels: all these columns follow the same filter strategy as the SNV columns for CaVEMan worksheet.
(XLSX)

S11 Table List of all filterd SNVs and indels found by cgpCaVEMan and cgpPindel. Chrom, pos: location of variant
Vartype: SNV or indel
Ref, alt: reference and alternate allele sequence of the variant
Variant allele fraction: alternate reads / reference reads for variant.
Validation result: If the variant coincides with the TrioDeNovo–called variant, then this variant was validated with MiSEQ. This column contains the validation result, if available.
(XLSX)

S12 Table Results for Kruskal-Wallis and Wilcoxon Rank Sum tests for cgpCaVEMan SNVs and cgpPindel indels. This table is organized in the same way as S7 Table, but shows tests on the final SNV and indel counts from S10 Table.
(XLSX)

## References

[1] Iyer V, Shen B, Zhang W, Hodgkins A, Keane T, Huang X, et al Off-target mutations are rare in Cas9-modified mice. Nature methods. 2015;12(6):479 10.1038/nmeth.3408 26020497

[2] Mianne J, Chessum L, Kumar S, Aguilar C, Codner G, Hutchison M, et al Correction of the auditory phenotype in C57BL/6N mice via CRISPR/Cas9-mediated homology directed repair. Genome medicine. 2016;8(1):16 10.1186/s13073-016-0273-4 26876963 PMC4753642

[3] Schaefer KA, Wu WH, Colgan DF, Tsang SH, Bassuk AG, Mahajan VB. Unexpected mutations after CRISPR-Cas9 editing in vivo. Nature methods. 2017;14(6):547–8. 10.1038/nmeth.4293 28557981 PMC5796662

[4] Le Fur N, Kelsall SR, Mintz B. Base substitution at different alternative splice donor sites of the tyrosinase gene in murine albinism. Genomics. 1996;37(2):245–8. 8921397 10.1006/geno.1996.0551

[5] Doe B, Brown E, Boroviak K. Generating CRISPR/Cas9-Derived Mutant Mice by Zygote Cytoplasmic Injection Using an Automatic Microinjector. Methods and Protocols. 2018;1(1):5. 10.3390/mps1010005 PMC6526459 31164552

[6] Pinello L, Canver MC, Hoban MD, Orkin SH, Kohn DB, Bauer DE, et al Analyzing CRISPR genome-editing experiments with CRISPResso. Nature biotechnology. 2016;34(7):695–7. 10.1038/nbt.3583 27404874 PMC5242601

[7] Li H. A statistical framework for SNP calling, mutation discovery, association mapping and population genetical parameter estimation from sequencing data. Bioinformatics (Oxford, England). 2011;27(21):2987–93. 10.1093/bioinformatics/btr509 PMC3198575 21903627

[8] Wei Q, Zhan X, Zhong X, Liu Y, Han Y, Chen W, et al A Bayesian framework for de novo mutation calling in parents-offspring trios. Bioinformatics (Oxford, England). 2015;31(9):1375–81. 10.1093/bioinformatics/btu839 PMC4410659 25535243

[9] Bae S, Park J, Kim JS. Cas-OFFinder: a fast and versatile algorithm that searches for potential off-target sites of Cas9 RNA-guided endonucleases. Bioinformatics (Oxford, England). 2014;30(10):1473–5. 10.1093/bioinformatics/btu048 PMC4016707 24463181

[10] Adewoye AB, Lindsay SJ, Dubrova YE, Hurles ME. The genome-wide effects of ionizing radiation on mutation induction in the mammalian germline. Nature communications. 2015;6:6684 10.1038/ncomms7684 25809527 PMC4389250

[11] Adams DJ, Doran AG, Lilue J, Keane TM. The Mouse Genomes Project: a repository of inbred laboratory mouse strain genomes. Mammalian genome: official journal of the International Mammalian Genome Society. 2015;26(9–10):403–12. 26123534 10.1007/s00335-015-9579-6

[12] Jones D, Raine KM, Davies H, Tarpey PS, Butler AP, Teague JW, et al cgpCaVEManWrapper: Simple Execution of CaVEMan in Order to Detect Somatic Single Nucleotide Variants in NGS Data. Current protocols in bioinformatics. 2016;56:15.0.1–.0.8. 10.1002/cpbi.20 PMC6097605 27930805

[13] Raine KM, Hinton J, Butler AP, Teague JW, Davies H, Tarpey P, et al cgpPindel: Identifying Somatically Acquired Insertion and Deletion Events from Paired End Sequencing. Current protocols in bioinformatics. 2015;52:15.7.1–2. 10.1002/0471250953.bi1507s52 PMC6097606 26678382

[14] Boroviak K, Doe B, Banerjee R, Yang F, Bradley A. Chromosome engineering in zygotes with CRISPR/Cas9. Genesis (New York, NY: 2000). 2016;54(2):78–85. 10.1002/dvg.22915 PMC4819711 26742453

[15] Wu W-H, Tsai Y-T, Justus S, Lee T-T, Zhang L, Lin C-S, et al CRISPR Repair Reveals Causative Mutation in a Preclinical Model of Retinitis Pigmentosa. Molecular Therapy. 2016;24(8):1388–94. 10.1038/mt.2016.107 27203441 PMC5023380

[16] Behringer RG, M.; Nagy, K.V.; Nagy, A. Manipulating the Mouse Embryo: A Laboratory Manual, Fourth Edition2014.

[17] Ryder E, Wong K, Gleeson D, Keane TM, Sethi D, Vyas S, et al Genomic analysis of a novel spontaneous albino C57BL/6N mouse strain. Genesis (New York, NY: 2000). 2013;51(7):523–8. 10.1002/dvg.22398 PMC3799019 23620107

