# Supplementary figures and images for: No unexpected CRISPR-Cas9 off-target activity revealed by trio sequencing of gene-edited mice
Source: PLoS Genet. 2018 Jul 9;14(7):e1007503. doi: 10.1371/journal.pgen.1007503 (PMC6057650; doi:10.1371/journal.pgen.1007503)

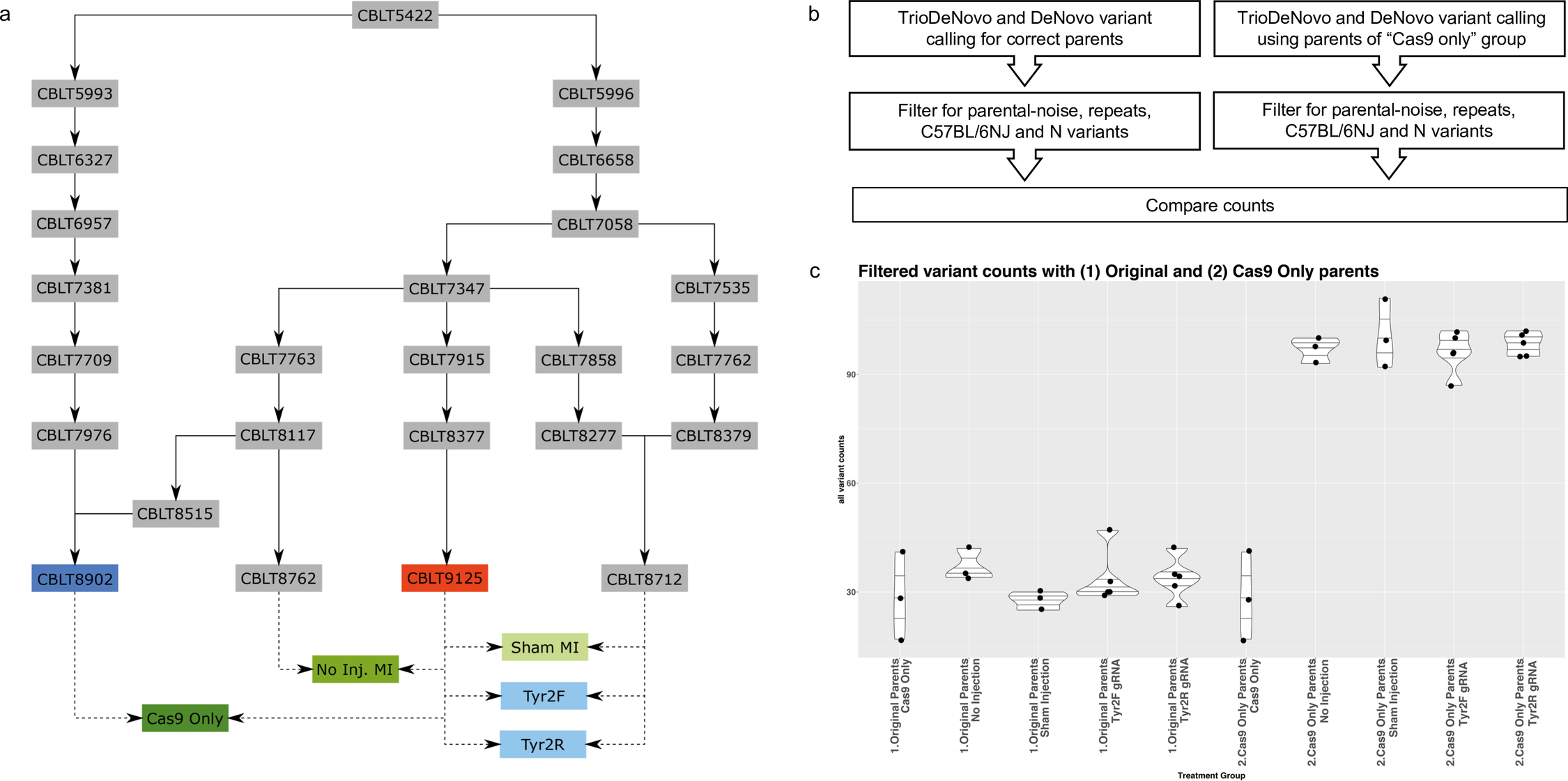

Supplement: S1 Fig — (a) Pedigree of heredity between the matings of mice from which mice parents were drawn. All female parents were drawn from mating CBLT9125 (red). Male parents were drawn from matings CBLT8712, CBLT8762 and CBLT8902 (dark blue). CBLT8712, CBLT8762 were greater than five generations distant from mating CBLT8902. (b) Scheme of the filtration pipelines for both the “correct” calling approach and the “incorrect parent” calling approach, generating variant counts that can be compared. (c) Graphs of variants counts from “correct parent” pipeline and “incorrect parent” pipeline showing the effect of choosing distantly related parents on de novo variant calls: an average increase of greater than 60 variants. (TIF) [file pgen.1007503.s001.tif]
